# Dramatic changes in malaria after the free distribution of mosquito nets in Papua New Guinea

**DOI:** 10.1186/1475-2875-11-S1-O46

**Published:** 2012-10-15

**Authors:** Manuel W Hetzel, Justin Pulford, Susan Paul, Nandao Tarongka, Hector Morris, Tony Tandrapah, Lisa Reimer, Leanne Robinson, Peter M Siba, Ivo Mueller

**Affiliations:** 1 Papua New Guinea Institute of Medical Research, Goroka, Papua New Guinea; 2 The University of Queensland, School of Population Health, Herston, Australia; 3 Case Western Reserve University, Center for Global Health and Disease, Cleveland, OH, USA; 4 Walter and Eliza Hall Institute of Medical Research, Melbourne, Australia; 5 Barcelona Centre for International Health Research (CRESIB, Hospital Clinic-Universitat de Barcelona), Barcelona, Spain

## Background

Papua New Guinea (PNG) is a South Pacific island nation with a complex malaria epidemiology. Four malaria species are transmitted by a variety of anopheline vectors filling the diverse ecological niches. Attempts to eliminate malaria in PNG in the 1950s -70s failed largely due to operational difficulties related to the unique implementation environment. Since 2004, the national malaria control program has been supported by two consecutive grants from the Global Fund resulting in the first country-wide free distribution of insecticide treated mosquito nets.

## Methods

Two cross-sectional household surveys carried out in 2008/09 and 2010/11 in randomly selected villages across PNG investigated changes in malaria control intervention coverage and population prevalence of malaria infection. Malaria surveillance in sentinel sites documented trends in the incidence of clinical cases and the prevalence of malaria infection among fever cases in health facilities. Prevalence of *Plasmodium* spp. was assessed by rapid diagnostic test (RDT) and light microscopy.

## Results

Country-wide household ownership of long-lasting insecticide treated nets (LLIN) reached 65% (n = 1958) in 2009 and over 80% (n = 1986) in 2011; usage in the target group of children under five years amounted to 40% (n = 1599) and over 55% (n=1768) in the respective years. Data from sentinel surveillance sites suggest that prior to the first LLIN distribution (2005 - 2009) both ownership and usage of LLIN were below 10%. No other malaria control interventions were introduced on a large scale during the mentioned period. Simultaneously, *Plasmodium* spp. prevalence in the general population decreased from 14% (n = 6442) in 2009 to below 7% in 2011 (n =7978). While the decrease was significant for *P. falciparum*, *P. vivax* parasite rates remained virtually unchanged resulting in a shift from *P. falciparum* to *P. vivax* dominanceinall regions. A significant decrease was also noted in malaria cases in sentinel health facilities where the proportion of fever cases with a positive RDT dropped from 56% pre-distribution (n = 1330) to 18% post-distribution (n = 681); however, the *P. falciparum* to *P. vivax* shift was less dramatic in clinical cases. Simultaneous entomological studies found a decrease in entomological inoculation rates but also short-term changes in biting behaviour.

## Conclusions

The dramatic effect of the Global Fund supported LLIN distribution on malaria in PNG poses new challenges to the national malaria control program. Implications for surveillance, prevention and treatment choices are discussed in consideration of experiences from comparable settings.

